# To the Editors of the Medical and Physical Journal

**Published:** 1802-02

**Authors:** W. White

**Affiliations:** Bath


### *To the Editors of the Medical and Phyfical Journal.*

GENTLEMEN,

FROM not reading the Medical and Phyfical Journal, for November, attentively, I did not perceive that you alluded to any remark I had made refpecting Bilious Diarrhœa. On referring however to what was ftated in the fubjoined note to the Lift for that that month, I acknowledge not to have expreſſed myſelf with ſufficient clearneſs. It no doubt has been obſerved in the Liſt that there were ſome caſes which I viewed as Idiopathic Dyſentery, becauſe not obviouſly connected with any morbid ſtate of the liver; in theſe caſes then it frequently happens that ſcybala are diſcharged. But in the Synochus Bilioſa Dyſenterica, where for the moſt part bile paſſes off in an increaſed quantity and vitiated ſtate, I believe ſcybala are very rarely perceived. In general alſo, in the latter affection, the quantity of blood is much greater than in the former, although attended with a leſs degree of teneſmus.

The Typhus Gravior, which I have mentioned in my account of the bilious fever juſt publiſhed, was at its acme the latter end of December, 1800, and diſappeared in January, 1801. I am, &c.

